# Incidence, prevalence, and outcome of primary biliary cholangitis in a nationwide Swedish population-based cohort

**DOI:** 10.1038/s41598-019-47890-2

**Published:** 2019-08-08

**Authors:** Hanns-Ulrich Marschall, Ida Henriksson, Sara Lindberg, Fabian Söderdahl, Marcus Thuresson, Staffan Wahlin, Jonas F. Ludvigsson

**Affiliations:** 1 Sahlgrenska Academy, Institute of Medicine, Department of Molecular and Clinical Medicine, University of Gothenburg, Gothenburg, Sweden; 2 Department of Medicine, Örebro University Hospital, Örebro, Sweden; 3 Department of Internal Medicine, Skaraborg Hospital, Skövde, 54142 Sweden; 4 Statisticon AB, Uppsala, Sweden; 5 Karolinska University Hospital Huddinge, Department of Gastroenterology and Hepatology, Stockholm, Sweden; 6 Department of Medical Epidemiology and Biostatistics, Karolinska Institutet, Stockholm, Sweden; 7 Department of Pediatrics, Örebro University Hospital, Örebro, Sweden

**Keywords:** Liver cirrhosis, Primary biliary cirrhosis

## Abstract

Available epidemiological data on primary biliary cholangitis (PBC) in Sweden originate from regional studies in the 1980s and may not reflect modern day PBC. We aimed to estimate incidence and prevalence, survival and death causes, and gender differences in PBC. We used international classification of disease (ICD) codes to identify patients with PBC in inpatient and outpatient registries 1987–2014 who were then linked to the Swedish cause of death, cancer and prescribed drug registries. Each PBC patient was matched with 10 reference individuals from the general population. In sensitivity analyses, we examined PBC patients identified through clinical patient records from Karolinska, Sahlgrenska and Örebro University Hospitals. We identified 5,350 adults with PBC. Prevalence of PBC increased steadily from 5.0 (1987) to 34.6 (2014) per 100,000 inhabitants whereas the yearly incidence rate was relatively constant with a median of 2.6 per 100,000 person-years, with a female:male gender ratio of 4:1. Compared to reference individuals, PBC individuals aged 15–39 years at diagnosis had a substantially higher risk of death (Hazard Ratio [HR] 12.7, 95% Confidence Interval [CI] 8.3–19.5) than those diagnosed between 40–59 (HR 4.1, 95% CI 3.7–4.5) and >60 (HR 3.7, 95% CI 3.5–3.9) years of age. Relative risks of mortality were highest in men. *In conclusion*, we found that recorded prevalence of PBC in Sweden has increased substantially during the last 30 years although incidence has been stable. Patients diagnosed in young adulthood were at a 12.7-fold increased risk of death, and male PBC patients had worse prognosis.

## Introduction

Primary Biliary Cholangitis (PBC) is a chronic immune-mediated liver disease characterized by progressive cholestasis, biliary fibrosis and eventually cirrhosis^1^. The disease was formerly known as Primary Biliary Cirrhosis, but this nomenclature was changed as only a minority of patients develop cirrhosis. About two out of three patients treated with ursodeoxycholic acid (UDCA) have an expected survival similar to the general population^2^. The pathogenesis of PBC remains unclear but the disease seems to be initiated by a combination of susceptible genetic background and exposure to environmental triggers, and as a result the prevalence varies between regions, with the highest incidence rates in northern Europe and Northern America^1^. The disease predominantly affects women and is typically diagnosed between 50 and 60 years of age^1^.

The majority of patients are asymptomatic at diagnosis and often PBC is suspected because of abnormal laboratory tests, particularly a raised serum alkaline phosphatase (AP) level. The diagnosis requires that at least 2/3 objective criteria are fulfilled; elevation of AP of liver origin for ≥6 months, elevation of serum anti-mitochondrial antibodies (AMA; titer ≥40) and characteristic histological features. Performing a liver biopsy is not necessary for the diagnosis if the two other criteria are present but is sometimes done for histological staging and/or to diagnose PBC with features of autoimmune hepatitis (AIH)^3^.

For almost twenty years, UDCA has been the standard and only approved treatment of PBC. It has been shown to prevent histologic progression and to improve survival without transplantation^3^. However, the response to UDCA varies, and unresponsive patients have a markedly worse prognosis. Regardless of biochemical response, UDCA does not seem to alleviate the cardinal symptoms of PBC: fatigue and pruritus^4^.

In recent years, much focus has been on finding optimal criteria to define UDCA responders and non-responders. A number of scoring systems have been established, in particular the dichotomized Barcelona^5^, Paris-1^6^, Rotterdam^7^, Toronto^8^, and Paris-2^9^ criteria. In 2015, the non-dichotomized GLOBE score was introduced, which is based on five variables: age at start of UDCA therapy, bilirubin, albumin, AP and platelet count at one year of follow-up^10^. In 2016, a similar scoring system was presented by UK-PBC that is based on baseline platelet count and serum albumin in combination with 1-year follow-up values of serum bilirubin, transaminases and AP^11^. The UK-PBC consortium and the Italian PBC group recently also developed a model that based on pretreatment variables is able to accurately predict UDCA response^12^.

All scoring systems aim to identify those patients who could benefit from therapies other than or in addition to UDCA but also those with a low risk of developing cirrhosis/end-stage liver disease and thus in no need of additional second-line therapy. Recently, obeticholic acid (OCA), a bile acid derivative that act on the nuclear farnesoid X receptor (FXR) was approved as second line therapy for patients not responding or being intolerant to UDCA^13^. Other agents such as peroxisome proliferator-activated receptor (PPAR) agonists may evolve as approved second line treatments^3^ and favorable results with bezafibrate have already been published^14^.

To underscore the need of novel treatment options, updated and robust data on the epidemiology and current management of PBC are needed also for Sweden as available information dates from regional studies in the 1980s^15–17^. This paper aims to provide these data by two complimentary approaches. First, by linking the Swedish inpatient and outpatient registries with the causes of death, cancer and prescribed drug registries, we estimate the contemporary (1) incidence and prevalence of PBC, (2) survival and death causes, (3) gender differences, (4) comorbidities, and (5) adherence to current treatment recommendations and potential second-lines treatments. Secondly, we validate these data from 2005, when the drug prescription registry was established, and provide information about UDCA response rates in different scoring systems, by comprehensively analyzing PBC patient files at Karolinska, Sahlgrenska and Örebro University Hospitals.

## Material and Methods

### Registry cohorts

A search was performed for adults (15 years of age and older) who had been assigned an International Classification of Diseases (ICD) code for PBC (ICD-9 (571G) until 1996 and/or ICD-10 (K743) from 1997) in the Swedish inpatient (1987–2014) or outpatient (2001–2014) registries. Extracted data were linked to the Swedish causes of death (1987–2014), cancer (1987–2014) and prescribed drug (July 2005–2014) registries (this latter register started in July 2005). We performed a search for liver diseases, liver-related complications and interventions, autoimmune and cardiovascular diseases, and cancer using relevant ICD-9 and ICD-10 codes, and PBC-specific medication by using Anatomic Therapeutic Chemical (ATC) classification codes as listed in Suppl. Table 1.

### Unexposed reference cohort

Each individual with PBC was matched for age, sex, calendar period and county with 10 reference individuals without a PBC diagnosis, by the government agency Statistics Sweden. Since this matching was done unintentially at first occurrence of any diagnosis, not necessarily PBC, some reference individuals were deceased at the time when the index individual was first diagnosed with PBC. Deceased reference individuals (n = 3365; 6.2% of the total reference group) were removed from further analyses.

### Verification cohort

All information about PBC patients (ICD-10 code K74.3) at Karolinska University Hospital Stockholm, Sahlgrenska University Hospital Gothenburg, and Örebro University Hospital was retrieved from the electronic clinical record file systems, electronic laboratory resources, and the electronic archives. The PBC diagnosis, i.e., fulfilment of at least 2/3 criteria (elevation of serum AP of liver origin for at least 6 months, elevation of serum anti-mitochondrial antibodies (AMA; titre ≥40) or characteristic histological features) was verified and information was gathered about time of diagnosis, PBC with features of AIH, other autoimmune disease, hepatocellular carcinoma, and time of liver transplantation or death. All patients with PBC diagnosed in 2005–2014 were selected for further assessment of medication and to evaluate the first year of UDCA treatment.

For every patient, UDCA treatment and all relevant variables for the seven scoring systems were recorded, such as sex, age (both current and at start of UDCA treatment), height, weight and BMI (calculated) at diagnosis, laboratory parameters at start of UDCA and at 1-year follow-up including bilirubin, ALT/AST, AP, albumin, platelet count, creatinine and PK-INR (using the spread sheet shown as Suppl. Table 2). When relevant data from the main hospital were missing, referring hospitals or caregivers were contacted by mail. In a few cases, where sufficient data were still not available, patients were excluded from all analyses.

All scores were calculated according to their original publications. Five of the scoring systems (Paris I, Paris II, Barcelona, Toronto, Rotterdam^5–9^) yield a dichotomized result, UDCA responder or non-responder, whereas the other two (UK-PBC risk score and GLOBE Score^10,11^) yield numeric scores and calculate risk or transplant-free survival at 3, 5, 10 and 15 years, respectively. For the Globe Score we also used a cut off value of 0.3 to define whether a patient is at high-risk for significantly shorter times of transplant-free survival or not^10^.

### Ethical approval

This study was approved, and individual informed consent was waived as this study was observational in nature^18^, by the regional ethics committees in Gothenburg (diary numbers, Dnr: 135–12, 870–15) and Uppsala (Dnr: 2016–262).

### Statistics

Incidence and prevalence were calculated using Swedish total population data from Statistics Sweden as a reference for each calendar year. Cox proportional hazards models were used to analyze differences in survival between the register cohort and the matched reference cohort. The models were stratified where each stratum contained one patient from the register cohort and the related matched reference individuals. Differences in comorbilities were tested using a 2-sample proportion test.

Chi square test was used to calculate differences between the register cohort and the verification cohort regarding sex and numbers treated. Since the verification cohort consists of patients from two large University Hospitals (Karolinska and Sahlgrenska) and one minor only recently approved University Hospital (Örebro), we compared these small cohorts internally using Chi square and Chi square for trends when appropriate. To calculate treatment response of individual biochemical variables, we used paired t-test. A p-value < 0.05 was considered statistically significant.

## Results

### Registry cohort

#### Incidence and prevalence

**Figure 1:**
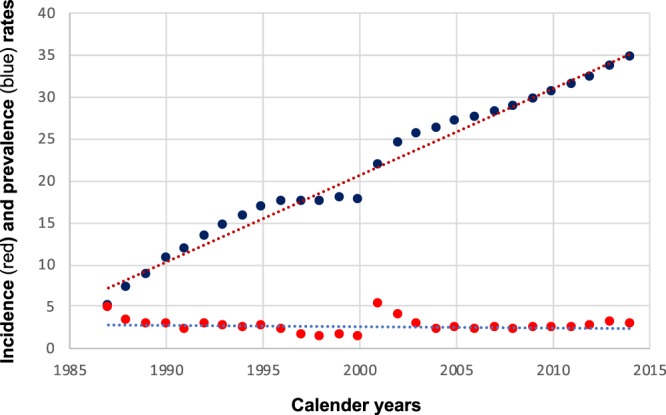
Incidence rates (per 100,000 person-years, red) and prevalence rates (per 100,000 person-years, blue) for PBC in Sweden from 1987 to 2015.

We identified 5,350 adults (15 years of age and older) with a PBC diagnosis and 50,145 reference individuals (Suppl. Table 3). The PBC incidence rate was around 2.6 per 100,000 person-years (Fig. 1) with some variability that mainly can be explained by the start of different registers (a likely overestimation 1987–1988, as well as 2001–2002), change in ICD-coding system and changes in care of patients. There was a steady increase in the registered prevalence of PBC from 5.0 (1987) to 34.6 (2014) per 100,000 inhabitants (Fig. 1 and Suppl. Table 4), also with some variability due to the difference in available registers. From 2004 to 2014 the data is however likely to cover the full prevalent cohort of PBC diagnosed patients, where the incidence is very stable, and the increase in prevalence is close to linear.

#### Gender relation

The median age of the whole study population was 64 years at diagnosis (range 15–94), and 9 out of 10 patients were born in Sweden (Suppl. Table 3). The female:male ratio in the whole study population was 4.0:1, with a higher proportion of women (5.4:1, 84.5%) in the outpatient population 2001–2014 (Suppl. Table 3).

#### Survival and liver events

**Figure 2:**
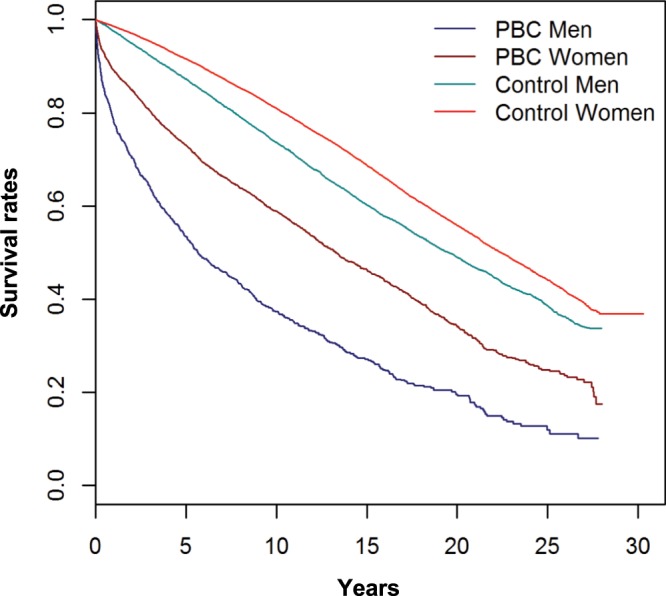
Kaplan-Meier plot for survival by gender and PBC.

Patients with PBC were at increased risk of death (Suppl. Table 6A). This was most evident for men of whom only 37.4%, in contrast to 58.9% of women, were alive 10 years after the first diagnosis of PBC, despite almost identical mean age at diagnosis (61.2 for men and 61.9 for women), as shown in the Kaplan-Meier plot (Fig. 2). The highest risk of death was observed in the first year after PBC diagnosis (Hazard Ratio, HR 9.0). The risk declined to a HR of 2.9 after more than 5 years (Suppl. Table 6A). Stratified by period of diagnosis, the risk constantly declined, from HR 5.6 during 1984–1989 to 3.0 to 2002–2015 (Suppl. Table 6B). Young age at diagnosis of PBC substantially increased the risk of death, with HR 12.7 at 15–39 years as compared to HR 4.1 between 40–59, and HR 3.7 from 60 years of age on (Suppl. Table 6C).

**Figure 3:**
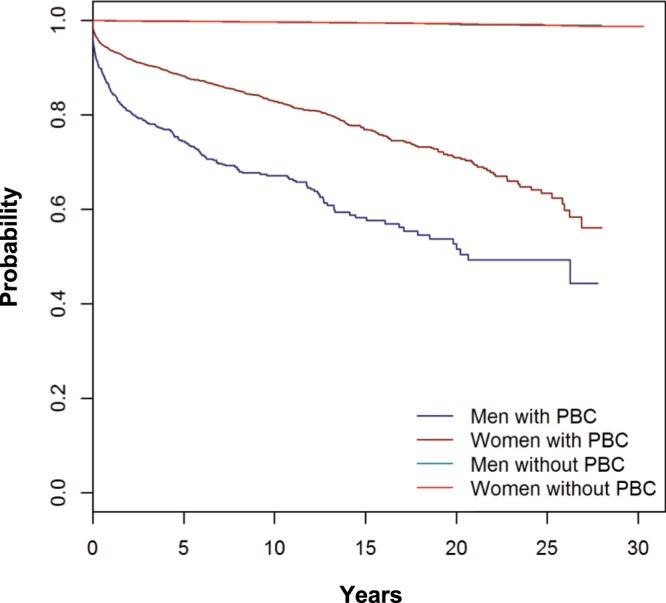
Kaplan-Meier plot for time to diagnosis of liver event. Liver event is defined as a esophageal varices, gastric varices, or ascites, including interventions for their treatment, and liver failure.

Significantly more men than women with PBC were diagnosed with liver complications such as esophageal varices and/or gastric varices, liver failure or ascites within ten years (32.9% vs. 17.1%, Fig. 3 and Suppl. Table 6D). Of note, survival analyses could not be stratified for whether or not the patients were treated with and responders to UDCA.

#### Causes of death

Patients with PBC had a significantly (p < 0.0001) increased risk of death from diagnoses related to digestive (HR, 22.85), cardiovascular (HR 1.43), cancer (HR 1.77), respiratory (HR 1.76) and other disease (HR 1.98) groups (Table 1). The major causes of death in PBC patients were digestive diseases related (22.9% vs. 2.5% in reference individuals), most of them liver related (13.8% vs. 0.2%, Table 2). Although, when hepatic complications such as liver cirrhosis, liver failure, esophageal or gastric varices, and ascites were removed from all digestive disease-related death diagnoses, relative mortality was still high (HR 8.8, Table 1). Since digestive and liver related death causes predominated in PBC patients, other common death causes were reported in relatively lower proportion, such as circulatory diseases (22.9% vs. 43.6%) and all cancers (18.7% vs. 25.4%), but hepatocellular cancer (HCC) was about 20 times more often cause of death as in reference individuals (4.1% vs. 0.2%, Table 2).

**Table 1:** Stratified Cox regression for cause specific deaths between PBC patients and reference individuals.

	Events PBC Patients	Events Reference Individuals	Group difference HR (95% CI)^#^	Group*time interaction HR (95% CI)
	(N = 5350)	(N = 50145)		
	n (%)	n (%)		
Digestive	629 (15.47)	415 (0.74)	22.85 (19.91–26.24)	1.03 (1.01–1.05)
Digestive w/o PBC	237 (5.83)	415 (0.74)	8.83 (7.45–10.47)	1.05 (1.03–1.07)
Cardiovascular	636 (15.65)	7133 (12.77)	1.43 (1.31–1.58)	1.01 (1.00–1.03)
Cancer	518 (12.74)	4193 (7.51)	1.77 (1.60–1.95)	1.00 (0.99–1.01)
Respiratory	100 (2.46)	937 (1.68)	1.76 (1.42–2.18)	1.04 (1.01–1.07)
Other	140 (3.44)	1050 (1.88)	1.98 (1.62–2.41)	0.99 (0.97–1.02)

As the hazard ration (HR) changes over time (see interaction HR) there is not one true HR regarding the treatment effect.

All group differences p < 0.0001; ^#^from year 2000 on.

**Table 2:** Causes of deaths and relative contribution to total mortality for deceased individuals during the study period.

	PBC Patients	Reference Individuals	Total
	(N = 2647)	(N = 15518)	(N = 18165)
	n (%)	n (%)	n (%)
**Cause of death**			
Circulatory	606 (22.89)	6770 (43.63)	7376 (40.61)
Digestive	607 (22.93)	381 (2.46)	988 (5.44)
Liver related	364 (13.75)	34 (0.22)	398 (2.19)
Endocrine and Metabolic	56 (2.12)	413 (2.66)	469 (2.58)
Respiratory	91 (3.44)	855 (5.51)	946 (5.21)
Infectious Disease	26 (0.98)	91 (0.59)	117 (0.64)
Musculoskeletal	18 (0.68)	70 (0.45)	88 (0.48)
Skin/subcutaneous	2 (0.08)	26 (0.17)	28 (0.15)
Neoplasms	495 (18.70)	3948 (25.44)	4443 (24.46)
CCC*	10 (0.38)	22 (0.14)	32 (0.18)
HCC*	109 (4.12)	28 (0.18)	137 (0.75)
Other	746 (28.18)	3338 (21.51)	4084 (22.48)

Note that no statistical comparison can be made until all patients and controls have died.

*CCC, cholangiocellular cancer; HCC, hepatocellular cancer.

#### Co-morbidities

At the time of diagnosis, patients with PBC compared to reference individuals had significantly (p < 0.001) more often a comorbidity diagnosis within all disease groups (Suppl. Table 7): infectious diseases (1.8% vs. 0.3%, in particular viral hepatitis), sarcoidosis (0.4% vs. 0.1%), endocrine and metabolic diseases (15.2% vs. 6.4%, in particular thyroid disease and diabetes mellitus, both type 1 and 2), circulatory diseases (28.0% vs. 14.4%, in particular essential hypertension) and among other than liver–related digestive diseases, Crohn’s disease (1.0% vs. 0.3%), ulcerative colitis (2.6% vs. 0.4%) and celiac disease (1.1% vs. 0.1%), There was also a higher prevalence of discoid lupus erythematosus (0.3% vs. 0.1%), psoriasis (2.2% vs. 0.9%) and a four-fold higher prevalence of musculoskeletal diseases (10.1% vs. 2.5%, in particular systemic lupus erythematosus and sicca/Sjögren syndrome).

Significant differences (p ≤ 0.001) for additional co-morbidities between PBC and reference individuals emerged throughout life-time follow-up (Suppl. Table 7), in particular for cancer: whereas liver cancer was more often found in patients with PBC than in reference individuals (4.4% vs. 0.3%), interestingly, it was the opposite for female cancer forms, breast cancer (3.8% vs. 5.1%), corpus uteri cancer (0.8% vs. 1.3%) and ovarian cancer (0.4% vs. 0.9%). Of note, there were no differences in any form of ischemic heart disease and actually a lower occurrence of cerebral infarction (5.8% vs. 7.1%). The diagnosis of primary sclerosing cholangitis (PSC) was not given to any reference individual but increased from 1.1% at baseline to 2.7% at any time in PBC patients, as did AIH, from 1.9% to 4.9%, during the course of PBC.

#### PBC related drug treatment and liver transplantation

Data for drug treatment were available from July 1, 2005. UDCA was administered as the recommended first line treatment to 79.9% of PBC patients (Suppl. Table 8), and usually within about a month after diagnosis, whereas second line treatments such as fibrates (0.5%) or budesonide (5.5%), including patients having PBC with features of AIH, were rare. Immunomodulators such as azathioprine, budesonide, 5-mercaptopurine and prednisolone were administered to at least 37.2% of PBC patients (Suppl. Table 8).

Liver transplantations for PBC were in Sweden performed from 1997 on, at a median number of 9.5 per year after PBC diagnosis (range, 4–14) (Suppl. Table 9). Related to the prevalence of PBC in Sweden, some 0.5% of the patients were transplanted annually, with no significant trend over time.

### Verification cohort characteristics and UDCA response rates

At Karolinska, Sahlgrenska and Örebro University Hospitals, 259 patients were diagnosed with incident PBC between 2004 and 2015 of which 231 patients with a mean age at diagnosis of 58.1 ± 1.6 years were suitable for UDCA response evaluation. Mean follow up was 6.4 ± 0.8 years, 200 (85.8%) were women (for additional characteristics see Table 3). Patients who did not fulfill the European Association for the Study of Liver (EASL) criteria for PBC^3^ (n = 8), who had insufficient data (n = 17) or had a follow up shorter than 12 months (n = 3) were excluded.

**Table 3:** Verification cohort: Gender; age at and years after diagnosis; treatment with and response rates to UDCA.

n	Karolinska	Sahlgrenska	Örebro	Total (mean ± SD, %)*
	122	78	31	231
Women	107 (87.7%)	67 (85.9%)	26 (83.9%)	200 (85.8%)
Age at diagnosis	58.1	56.5	59.7	58.1 ± 1.6
Years with PBC	7.1	6.6	5.6	6.4 ± 0.8
UDCA	107 (87.7%)	68 (87.2%)	19 (61.3%)	194 (78.7 ± 15.1%)
Paris 1	91 (74.6%)	52 (76.5%)	15 (78.9%)	73.3 ± 7.7%
Paris 2	76 (62.3%)	39 (57.4%)	11 (57.9%)	59.2 ± 2.7%
Barcelona	85 (69.7%)	42 (61.8%)	14 (73.7%)	68.4 ± 6.1%
Toronto	87 (71.3%)	41 (60.3%)	12 (63.2%)	64.9 ± 5.7%
Rotterdam	74 (60.7%)	47 (69.1%)	10 (52.6%)	60.8 ± 8.3%

*mean ± SD of cohorts.

UDCA was prescribed to 194 (84.0%) of all 231 patients, with no differences between women and men. Younger patients tended to be prescribed UDCA more often than elder ones: about 95.0% of those diagnosed with PBC between 18–39 years of age compared to 85.7 of those diagnosed between 40–59 and 80.2% of those over 60 years, p = 0.086 (adjusted for sex and age at diagnosis).

The verification cohort demonstrated higher AP levels (5.1 x upper limit normal (ULN), 3.0–7.5; median, interquartile range)) than the large cohorts of the GLOBE score^10^ (2.11 x ULN; 1.37–3.79) and UK-PBC Risk Score^11^ (1.9 x ULN; 1.2–3.5). AP but not transaminase levels changed significantly after one year of UDCA treatment. The median AP levels decreased from 5.1 x ULN (3.0–7.5) to 2.9 x ULN (1.7–5.6) (p < 0.0001), which is a reduction by 43.1%.

Overall treatment response rates varied between 73.3 ± 7.7% % for Paris 1 and 59.2 ± 2.7% % for Paris 2 criteria, with the other criteria in between, with no significant differences between Hospitals (Table 3). By summarizing all five dichotomized systems, 82 (42.3%) of all 194 UDCA-treated patients were unanimously identified as responders, and only 9 (4.6%) as non-responders. Additional 36 patients were UDCA-responders according to 4, and 10 according to 3 scoring systems.

For the GLOBE Score, separate cut off values were defined for different age groups to define High Risk patients regardless of age. In our material 26 (13.4%) patients were defined as High Risk patients. The UK-PBC risk score was calculated for 158 of the 194 treated patients (the data set was incomplete for 38 patients). Median hazard ratios and IQRs of a liver event (liver death or liver transplantation) were: after 5 years; 0.71 (0.41–1.18), after 10 years, 2.34 (1.36–3.90), and after 15 years, 4.32 (2.51–7.14).

## Discussion

This nationwide population-based study found a prevalence rate of PBC in Sweden of about 35 per 100,000 inhabitants at the end of the observation period. This is substantially higher than the 9.2–15.1 per 100,000 reported >30 years ago and based on only 162 patients^15–17^. Our study included 5,350 adult PBC cases and the prevalence is among the highest reported worldwide^19^, higher than in Danmark but close to Iceland (19.0 and 38.3/100,000 inhabitants, respectively, in 2009/2010^20,21^). We found a female to male ratio of 4.0:1, which is almost identical to that in Denmark (4.2:1)^21^. As we did not observe a significant trend in the incidence rates between 1987 and 2014, the linearly increasing prevalence rates argue against influence of practice changes regarding input of data into the registries during the time period.

Our data do not show significantly increased overall survival since the introduction of UDCA in the Swedish health care in 1998. UDCA is now considered as recommended first line treatment for PBC patients. However, 20.1% of Swedish PBC patients were not treated with UDCA, at least during the last 10 years of follow-up when prescribed drug data is available, which is in line with a recently reported rate of UDCA intolerance^13^. We were not able to stratify overall mortality or clinical disease progression according to UDCA response, since these data were unavailable in our registry cohort. Second line treatment of PBC according to previous EASL guidelines, i.e., fibrates or budesonide, was rare^22^. Immunomodulators, such as azathiopurine, budesonide, 5-mercaptopurine and prednis(ol)one were most likely administered for the treatment of other immune-mediated endocrine, gastrointestinal, musculoskeletal and skin diseases rather than true AIH-PBC overlap.

Indeed, our registry study confirms a strong association of PBC with other immune-mediated endocrine, musculoskeletal and skin diseases such as thyroid disease, sicca syndrome, discoid and systemic lupus erythematosus, but also with inflammatory bowel diseases such as Crohn’s disease and ulcerative colitis. Of note, our registry study found that 1.1% of PBC patients also had a diagnosis of celiac disease. This percentage is lower than previously reported (3.1%^23^). The finding of 2.7% of PSC among the PBC-patients in the registry cohort, is somewhat troublesome, especially since overlap PBC-PSC is rare, and all earlier research is based solely on case-reports. Most probably this reflects misdiagnosis.

Consistent with UK data^24^, Swedish PBC patients are at increased risk of death, with an excess mortality especially in men. Men were in the early years also over-represented among the inpatients. As expected, we found an excess mortality due to liver-related deaths. PBC patients had overall lower cancer mortality despite a substantially increased risk of HCC. Our finding of a 19% overall cancer mortality is higher than old data from Sweden (12%)^25^ but in perfect agreement with a recent meta-analysis that found both an increased overall risk for cancer (pooled rate ratio, RR, 1.55) and for HCC (pooled RR 18.8) in PBC but not for other cancers^26^.

Our verification cohort shows similar characteristics to PBC cohorts around the world^5–11^, and confirmed registry findings for 2005–2014 regarding gender ratio and adherence to treatment with UDCA. Between 15% and 20% of PBC patients are male and only about 80% of PBC patients are treated with UDCA. A bias might be introduced by the low UDCA prescription rate of 61.3% for PBC patients at Örebro University Hospital but still, treatment rates of 87.5% at Karolinska and Sahlgrenska University Hospitals were less than expected.

We developed a spread sheet for clinical and biochemical data that was easy to use and immediately reported UDCA response in all major PBC scoring systems (Suppl. Table 2). Our verification cohort demonstrated higher AP levels than the large cohorts of the GLOBE^10^ and UK-PBC Risk Scores^11^, but so did also the cohorts used for the Paris 1^6^, Paris 2^9^ and Barcelona^5^ criteria. We observed a decrease of AP levels after one year of treatment with UDCA of 43.1%, which is about the same as the decreases of 45% and 49%, respectively, in the Paris-1 and Paris-2 cohorts, respectively, and more than the decreases of 36% and 37%, respectively, in the GLOBE and UK-PBC cohorts. Treatment response rates in our verification cohort were between 59.2% (Paris 2) and 73.3% (Paris 1). This is similar to earlier findings that up to 30% show inadequate biochemical response to UDCA (rising to >50% in patients presenting under the age of 40 years)^24^. In the UK cohort, 80% of patients responded to treatment according to the Paris-1 criteria^24^, a figure similar to the 73.3% in our verification cohort, further supporting the use of at least this easy-to-use criterion in clinical practice, in particular outside specialized centers. Of note, Paris 2 criteria have been designed specifically to better fit early-stage patients^9^ but disease stage information was not available from our verification cohort. Otherwise Paris 1 is recommended, as this score evaluates the two most important biochemical parameters, bilirubin and AP. Sereval studies have found that this score best discriminates between low-risk and high-risk PBC patients^22^.

Smartphone applications may in the near future allow easy application of more complex algorithms to estimate outcome^10,11^ and potentially even without awaiting UDCA response^12^ and thus ensure timely treatment^27^. This has important consequences both for the individual patient, in choosing candidates for second line treatment, for future research and for health economic purposes as patients with a good prognosis could be managed at a primary care level.

## Strengths and Limitations

In this nationwide population-based study spanning almost 30 years of follow-up we evaluated more than 5000 individuals with PBC. Through the unique personal identity number^28^ we were able to link study participants to age- and sex-matched reference individuals for comparisions but also to major healthcare registers. The personal identity number of all participants also guarantees a virtually complete follow-up. We used the Swedish Patient Register to identify PBC. While we are unaware of any validation of PBC, the positive predictive value for most chronic diagnoses in this register is 85–95%^29^. The current study was not designed to examine sensitivity of the in-patient vs. out-patient-registries per se, but this has been looked at before^30^. Swedish healthcare is tax-funded and this should rule ut major selection bias due to socioeconomic status^30^. Finally, we were able to verify our data using an extensive verification cohort from three different university clinics. Among the weaknesses is the fact that the Patient Register only include outpatient care since 2001 and this may explain the initial increase in prevalence and incidence after 2001. Also, we were only able to examine UDCA treatment since 2005, because the Prescribed Drug Register started in this year.

## Conclusion

In conclusion, this nationwide population-based cohort found a stable incidence but rising prevalence of PBC in Sweden, with a much higher proportion of men than reported earlier. PBC has a substantial morbidity and mortality, especially from liver related causes. First-line therapy, and where UDCA fails second-line treatment, are crucial for patient management.

## Supplementary information

Supplementary Tables 1–9

## References

[1] Carey EJ, Ali AH, Lindor KD (2015). Primary biliary cirrhosis. Lancet.

[2] Beuers U (2015). Changing nomenclature for PBC: From ‘cirrhosis’ to ‘cholangitis’. J. Hepatol..

[3] Liver EAFTSOT (2017). EASL Clinical Practice Guidelines: The diagnosis and management of patients with primary biliary cholangitis. J. Hepatol..

[4] Dyson JK (2015). Novel therapeutic targets in primary biliary cirrhosis. Nat. Rev. Gastroenterol. Hepatol..

[5] Pares A, Caballeria L, Rodes J (2006). Excellent long-term survival in patients with primary biliary cirrhosis and biochemical response to ursodeoxycholic Acid. Gastroenterology.

[6] Corpechot C (2008). Biochemical response to ursodeoxycholic acid and long-term prognosis in primary biliary cirrhosis. Hepatology.

[7] Kuiper EM (2009). Improved prognosis of patients with primary biliary cirrhosis that have a biochemical response to ursodeoxycholic acid. Gastroenterology.

[8] Kumagi T (2010). Baseline ductopenia and treatment response predict long-term histological progression in primary biliary cirrhosis. Am. J. Gastroenterol..

[9] Corpechot C, Chazouilleres O, Poupon R (2011). Early primary biliary cirrhosis: biochemical response to treatment and prediction of long-term outcome. J. Hepatol..

[10] Lammers WJ (2015). Development and Validation of a Scoring System to Predict Outcomes of Patients With Primary Biliary Cirrhosis Receiving Ursodeoxycholic Acid Therapy. Gastroenterology.

[11] Carbone M (2016). The UK-PBC risk scores: Derivation and validation of a scoring system for long-term prediction of end-stage liver disease in primary biliary cholangitis. Hepatology.

[12] Carbone Marco, Nardi Alessandra, Flack Steve, Carpino Guido, Varvaropoulou Nikoletta, Gavrila Caius, Spicer Ann, Badrock Jonathan, Bernuzzi Francesca, Cardinale Vincenzo, Ainsworth Holly F, Heneghan Michael A, Thorburn Douglas, Bathgate Andrew, Jones Rebecca, Neuberger James M, Battezzati Pier Maria, Zuin Massimo, Taylor-Robinson Simon, Donato Maria F, Kirby John, Mitchell-Thain Robert, Floreani Annarosa, Sampaziotis Fotios, Muratori Luigi, Alvaro Domenico, Marzioni Marco, Miele Luca, Marra Fabio, Giannini Edoardo, Gaudio Eugenio, Ronca Vincenzo, Bonato Giulia, Cristoferi Laura, Malinverno Federica, Gerussi Alessio, Stocken Deborah D, Cordell Heather J, Hirschfield Gideon M, Alexander Graeme J, Sandford Richard N, Jones David E, Invernizzi Pietro, Mells George F, Thomas Caradog, Rahman Meshbah, Yapp Tom, Lye Ch'ng Chin, Harrison Melanie, Sturgess Richard, Galaska Roman, Healey Chris, Whiteman Jessica, Czaijkowski Marek, Gray Catherine, Gunasekera Anton, Gyawli Pranab, Premchand Purushothaman, Mann Steven, Elliott Keith, Kapur Kapil, Watson Alan, Foster Graham, Trembling Paul, Subhani Javaid, Harvey Rory, McCorry Roger, Adgey Carolyn, Hobson Lucie, Mulvaney-Jones Caroline, Evans Richard, Mathialahan Thiriloganathan, Ramanaden David, Gasem Jaber, Van Duyvenvoorde Greta, Shorrock Christopher, Seward Katie, Southern Paul, Tibble Jeremy, Penn Ruth, Gorard David, Maiden Jane, Damant Rose, Palegwala Altaf, Jones Susan, Alexander Graeme, Mells George, Sandford Richard, Whiteman Jessica, Dolwani Sunil, Prince Martin, Silvestre Valeria, Foxton Matthew, Dungca Eleanor, Mitchison Harriet, Wheatley Natalie, Gooding Ian, Doyle Helen, Karmo Mazn, Kent Melanie, Saksena Sushma, Braim Delyth, Patel Minesh, Lord Susan, Ede Roland, Paton Alison, Austin Andrew, Lancaster Nicola, Sayer Joanna, Gibbins Andrew, Hogben Karen, Hovell Chris, Fisher Neil, Carter Martyn, Koss Konrad, Musselwhite Janine, Muscariu Florin, Piotreowicz Andrzej, McKay Alexandra, Grimley Charles, Neal David, Ting Tan Lai, Lim Guan, Brighton Jacqueline, Foale Carole, Ala Aftab, Saeed Athar, Flahive Kerry, Wood Gordon, Townshend Paula, Ford Chris, Brown Jonathan, Kordula Jean, Bowles Jane, Wilkinson Mark, Palmer Caroline, Ramage John, Gordon Harriet, Featherstone James, Ridpath Jo, Ngatchu Theodore, Levi Sass, Shaukat Syed, Sadeghian Joy, Shidrawi Ray, Williams Bronwen, Abouda George, Jones Sarah, Duggan Claire, Hynes Abigail, Narain Mark, Rees Ian, Salam Imroz, Crossey Mary, Taylor-Robinson Simon, Brown Ashley, MacNicol Carolyn, Williams Simon, Wilhelmsen Elva, Banim Paul, Raymode Parizade, Chilton Andrew, Das Debasish, Lee Hye-Jeong, Curtis Howard, Heneghan Michael, Gess Markus, Durant Emma, Drake IM, Bishop Rebecca, Davies Mervyn, Jones Rebecca, Aldersley Mark, Ncube Noma, McNair Alistair, Srirajaskanthan Raj, Sen Sambit, Casey Rebecca, Bird George, Mendall Mike, Cowley Caroline, Barnardo Adrian, Kitchen Paul, Yoong Kevin, Amore Kelly, Sirdefield Dawn, Orpe Jacky, Mathew Ray, MacFaul George, Wrigth Aruna, Shah Amir, Evans Chris, Keggans Janie, Bird Bridget, Baxter Gwen, Saha Subrata, Pollock Katharine, Hughes Maggie, Bramley Peter, Grieve Emma, Young Karin, Fraser Andrew, Mukhopadhya Ashis, Ocker Kate, Mills Peter, Hines Francis, Shallcross Chris, Wilkins Joy, Grellier Leonie, Campbell Stewart, Martin Kirsty, Bathgate Andrew, Innes Caron, Shepherd Alan, Rushbrook Simon, Valliani Talal, Przemioslo Robert, Fairlamb Helen, Macdonald Chris, Eastick Anne, Metcalf Jane, Tanqueray Elizabeth, Shmueli Udi, Holbrook Becky, Davis Andrew, Browning Julie, Naqvi Asifabbas, Walker Kirsten, Lee Tom, Verheyden Juliette, Slininger Susan, Ryder Stephen D, Chapman Roger, Collier Jane, O'Donnell Denise, Stafford Lizzie, Williamson Kate, Kent Linda, Klass Howard, Ninkovic Mary, March Linda, Cramp Matthew, Simpson Diane, Dickson Christine, Sharer Nicholas, Hayes Maria, Goggin Patrick, Quinne Mary, Pearson Sallyanne, Hoeroldt Barbara, Jones Linda, Wright Alice, Booth Jonathan, Loftus Alison, Lipscomb George, Dewhurst Hannah, Gunter Emma, Williams Earl, Fouracres Anna, Farrington Liz, Graves Lyn, Hussaini Hyder, Stableforth Bill, Marriott Suzie, Ayres Reuben, Leoni Marina, Burroughs Andrew, Marshall Eileen, Thorburn Douglas, Tyrer David, Martin Kate, Lombard Martin, Patanwala Imran, Dali-Kemmery Lola, Lambourne Victoria, Maltby Julia, Vyas Samir, Colley Julie, Shinder Bal, Singhal Saket, Jones Jayne, Mills Marisa, Gleeson Dermot, Carnahan Mandy, Butterworth Jeff, Boulton Kerenza, Taylor Natalie, George Keith, Harding Tim, Tregonning Julie, Douglass Andrew, Brown Carly, Clifford Gayle, Panter Simon, Gocher Denise, Shearman Jeremy, Bray Gary, Hamilton Maria, Butcher Graham, Forton Daniel, Mclindon John, Curtis Janette, Das Debashis, Shewan Tracey, Cowan Matthew, Whatley Gregory, Nasseri Mariam, Grover Bob, Sivaramakrishnan Nurani, Ducker Samantha, Houghton Kathryn, Jones David, Griffiths Laura, Tripoli Sherill, Pitcher Maxton, Shpuza Ervin, White Nikki, Ghosh Deb, Douds Andrew, Green Marie, Brookes Matthew, Cumlat Lourdes, Wong Voi Shim, Warner Karen, Netherton Kimberley, Mandal Adtya, Jain Snjiv, Gupta Hemant, Sanghi Pradeep, Pereira Steve, Neuberger James, Gunson Bridget, Hirschfield Gideon, Lim Reina Teegan, Gallagher Susan, Clement Darren, Brind Alison, Watts Gill, Mupudzi Mcdonald, Wright Mark, Gitahi Jane, Gordon Fiona, Gocher Denis, Unitt Esther, Pateman Hilary, Batham Sally, Delahooke Toby, Grant Allister, Conder Jill, Higham Andrew, Cox Mark, O'Donohoe Lynn, Currie Lynn, King Alistair, Oblak Metod, Collins Carole, Whalley Simon, Quinn Marie, Baird Yolanda, Amey Isobel, Fraser Jocelyn, Li Andy, Cotterill Donna, Bell Andrew, Watson Alan, Singhal Amit, Gee Ian, Greer Sandra, Ang Yeng, Ransford Rupert, Allison Joanna, Gotto James, Dyer Simon, Sweeting Helen, Millson Charles, Invernizzi Pietro, Carbone Marco, Cristoferi Laura, Bonato Giulia, Malinverno Federica, Bernuzzi Francesca, Alvaro Domenico, Labbadia Giancarlo, Bragazzi Maria Consiglia, Andreone Pietro, Muratori Luigi, Azzaroli Francesco, Floreani Annarosa, Galli Andrea, Tarocchi Mirko, Giannini Edoardo, Miele Luca, Gasbarrini Antonio, Grieco Antonio, Marrone Giuseppe, Donato Maria Francesca, Valenti Luca, Marra Fabio, Marzioni Marco, Maroni Luca, Rigamonti Cristina, Zuin Massimo, Battezzati Pier Maria, Picciotto Antonino (2018). Pretreatment prediction of response to ursodeoxycholic acid in primary biliary cholangitis: development and validation of the UDCA Response Score. The Lancet Gastroenterology & Hepatology.

[13] Nevens F (2016). A Placebo-Controlled Trial of Obeticholic Acid in Primary Biliary Cholangitis. N. Engl. J. Med..

[14] Corpechot C (2018). A Placebo-Controlled Trial of Bezafibrate in Primary Biliary Cholangitis. N. Engl. J. Med..

[15] Eriksson S, Lindgren S (1984). The prevalence and clinical spectrum of primary biliary cirrhosis in a defined population. Scand. J. Gastroenterol..

[16] Lofgren J, Jarnerot G, Danielsson D, Hemdal I (1985). Incidence and prevalence of primary biliary cirrhosis in a defined population in Sweden. Scand. J. Gastroenterol..

[17] Danielsson A, Boqvist L, Uddenfeldt P (1990). Epidemiology of primary biliary cirrhosis in a defined rural population in the northern part of Sweden. Hepatology.

[18] Ludvigsson JF (2016). Registers of the Swedish total population and their use in medical research. Eur. J. Epidemiol..

[19] Boonstra K, Beuers U, Ponsioen CY (2012). Epidemiology of primary sclerosing cholangitis and primary biliary cirrhosis: a systematic review. J. Hepatol..

[20] Baldursdottir TR (2012). The epidemiology and natural history of primary biliary cirrhosis: a nationwide population-based study. Eur. J. Gastroenterol. Hepatol..

[21] Lleo A (2016). Evolving Trends in Female to Male Incidence and Male Mortality of Primary Biliary Cholangitis. Sci. Rep..

[22] EASL Clinical Practice Guidelines: management of cholestatic liver diseases. *J. Hepatol*. **51**, 237–267, S0168-8278(09)00309-2 [pii], 10.1016/j.jhep.2009.04.009 (2009). 10.1016/j.jhep.2009.04.009 19501929

[23] Volta U, Caio G, Tovoli F, De Giorgio R (2013). Gut-liver axis: an immune link between celiac disease and primary biliary cirrhosis. Expert Rev. Gastroenterol. Hepatol..

[24] Carbone Marco, Mells George F., Pells Greta, Dawwas Muhammad F., Newton Julia L., Heneghan Michael A., Neuberger James M., Day Darren B., Ducker Samantha J., Sandford Richard N., Alexander Graeme J., Jones David E.J. (2013). Sex and Age Are Determinants of the Clinical Phenotype of Primary Biliary Cirrhosis and Response to Ursodeoxycholic Acid. Gastroenterology.

[25] Loof L (1994). Cancer risk in primary biliary cirrhosis: a population-based study from Sweden. Hepatology.

[26] Liang Y, Yang Z, Zhong R (2012). Primary biliary cirrhosis and cancer risk: a systematic review and meta-analysis. Hepatology.

[27] Marschall Hanns-Ulrich (2018). Ensuring timely treatment of patients with primary biliary cholangitis. The Lancet Gastroenterology & Hepatology.

[28] Ludvigsson JF, Otterblad-Olausson P, Pettersson BU, Ekbom A (2009). The Swedish personal identity number: possibilities and pitfalls in healthcare and medical research. Eur. J. Epidemiol..

[29] Ludvigsson JF (2011). External review and validation of the Swedish national inpatient register. BMC Public Health.

[30] Anell A (2015). The public-private pendulum–patient choice and equity in Sweden. N. Engl. J. Med..

